# A smartphone-guided secondary prevention digital health application reduces systolic blood pressure in patients with chronic coronary syndrome and insufficient blood pressure control

**DOI:** 10.3389/fcvm.2025.1515598

**Published:** 2025-02-10

**Authors:** Philip Düsing, Stephan H. Schirmer, Sebastian Schäfer, Alexander Krogmann, Jan-Malte Sinning, Nikos Werner, Florian Bönner, Alexander Sedaghat, Cornelius Müller, Irina Eckardt, Georg Nickenig, Andreas Zietzer

**Affiliations:** ^1^ Department of Medicine II, Heart Center, University Hospital Bonn, Bonn, Germany; ^2^ Kardiopraxis Schirmer, Kaiserslautern, Germany; ^3^ Praxis Rheingalerie Rodenkirchen, Cologne, Germany; ^4^ Kardio-Lev, Kardiologische Gemeinschaftspraxis Dr. P. Son, Dr. M. Päsler, Dr. A. Krogmann, Leverkusen, Germany; ^5^ Department of Cardiology, St. Vinzenz Hospital, Cologne, Germany; ^6^ Medical Department III, Heart Center Trier, Krankenhaus der Barmherzigen Brüder, Trier, Germany; ^7^ Department of Cardiology, Pulmonology, and Vascular Medicine, Medical Faculty of Heinrich Heine University, University Hospital Düsseldorf, Düsseldorf, Germany; ^8^ Rhein-Ahr-Cardio, Praxis für Kardiologie, Bad Neuenahr-Ahrweiler, Germany; ^9^ Kardio Bonn, Gemeinschaftspraxis Dr. La Rosée & Prof. Müller, Bonn, Germany

**Keywords:** chronic coronary syndrome, secondary prevention, digital health, hypertension, smartphone

## Abstract

**Background:**

Chronic coronary syndrome (CCS) leads to high morbidity and mortality despite therapeutic advances in recent decades. Several modifiable risk factors, including increased blood pressure (BP), significantly contribute to cardiovascular risk in CCS. Therefore, optimal secondary prevention includes managing BP through lifestyle changes and pharmacological therapy. The CHANGE study aimed to provide evidence for optimizing secondary prevention in CCS patients using a smartphone application.

**Methods:**

The CHANGE-Study is a prospective, randomized, controlled trial performed in 9 centers in Germany. Patients with CCS were randomly allocated to either a control or an intervention group. The intervention group received the “Vantis | KHK und Herzinfarkt” digital health application and standard care. The control group received standard care alone. From the original cohort, subgroups of patients with systolic BP ≥140 mmHg (*n* = 44), ≥130 mmHg (*n* = 89) and diastolic BP ≥90 mmHg (*n* = 28) were analyzed for BP reduction after 12 weeks.

**Results:**

In patients with systolic BP ≥140 mmHg, the intervention group showed a reduction in systolic BP by 15.5 mmHg (± 16.7 mmHg, *p* = 0.0001), which was greater compared to the control group (6.0 ± 13.0 mmHg, *p* = 0.058). This observation was consistent in patients with systolic BP ≥130 mmHg at baseline. No significant differences between both groups were observed in diastolic BP reduction in patients with diastolic BP ≥90 mmHg.

**Conclusion:**

The CHANGE study documents that a smartphone-guided digital health application positively affects systolic BP in CCS patients. This study underlines the potential of digital interventions in cardiology to improve secondary prevention.

## Graphical Abstract

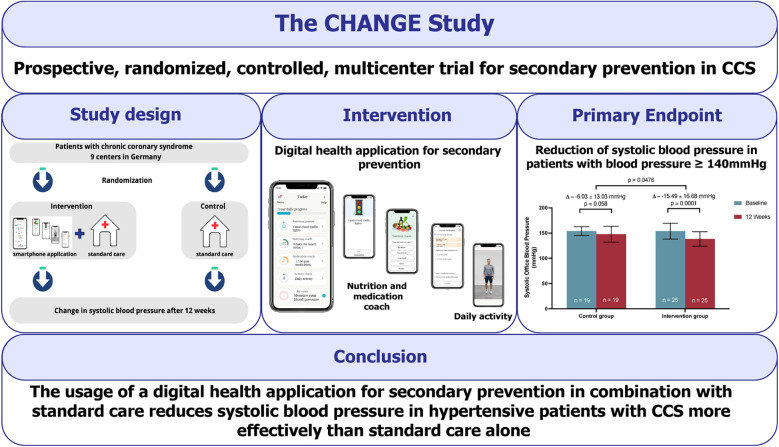

## Background

Coronary artery disease (CAD) is the manifestation of atherosclerosis in the coronary arteries. The pathogenesis of CAD is characterized by chronic inflammation, endothelial dysfunction and progressive atherosclerotic plaque formation (1, 2). Clinical presentations of CAD can be categorized as acute and chronic coronary syndromes (CCS) (2). CCS, formerly called stable CAD, is a dynamic state markedly influenced by cardiovascular risk factors such as hypertension, dyslipidemia and diabetes (2). Further, lifestyle factors such as smoking, diet, abdominal obesity, and lack of physical activity influence cardiovascular risk significantly (3, 4). These factors combined are known as “standard modifiable risk factors”, and among these, hypertension is highly prevalent and aggressive (5, 6). The *Global burden of Disease study* identified elevated blood pressure (BP) as the leading risk factor for death (7). Thus, BP control is crucial, especially in high-risk populations such as CCS patients. Current ESC guidelines on CCS recommend systolic BP to be targeted at 120–129 mmHg in most patients if the treatment is well tolerated (2, 8).

CAD remains the number one cause of death despite significant therapeutic advances in the past decades (9). Numerous epidemiological and interventional studies demonstrate that control of modifiable risk factors is associated with a significantly improved prognosis in patients with cardiovascular disease (10). However, morbidity and mortality among patients with CCS remain high (9). It is generally agreed that insufficient management of modifiable risk factors plays a significant role in this context. Critical issues in risk factor management include poor adherence to medical therapy, lifestyle advice and insufficient behavioural change (11–13). Consequently, evidence-based recommendations addressing adherence and lifestyle management are provided in several guidelines (2, 14–16). Physicians are advised to address such issues at every clinical appointment (2). However, implementing this practice is challenging due to prolonged gaps between clinical appointments and high turnover patient settings (17).

The field of digital cardiology is experiencing rapid growth. In recent years, smartphone apps have emerged as promising strategies to enhance cardiovascular care. A meta-analysis including 5165 patients with cardiovascular disease showed that patients using mobile health approaches, including text messaging and mobile apps, had increased adherence to medical therapy and the ability to reach blood pressure targets and exercise goals (2, 18). Among others, these data have resulted in mobile health interventions being recommended to improve patient adherence in current ESC guidelines on CCS (2). Despite these promising signals, several international studies investigating various digital interventions have published controversial results (19–21). No evidence is available from studies investigating the influence of digital interventions in a European cohort of CCS patients. In addition, no randomized trials have investigated apps with monitoring and therapeutic features in such patients. The CHANGE study aimed to provide such evidence for secondary prevention in patients with CCS using a smartphone digital health application.

## Methods

### Study design

The CHANGE study (A prospective, randomized, controlled, multicenter trial for secondary prevention in patients with CCS using a smartphone application for digital therapy) was devised as a prospective, randomized, controlled trial with a 1:1 allocation ratio in a parallel group design that was performed in 9 centers in Germany. The study rationale and design have been published previously (22). Patients with CCS were randomly allocated to either the control or the intervention group. Patients in the intervention group were given the “Vantis | KHK und Herzinfarkt” digital health application combined with a blood pressure monitor and standard care. The control group received standard care alone. This comprised appropriate pharmacological therapy, periodical assessment by a cardiovascular caregiver (cardiologist, internist, general practitioner, or cardiovascular nurse) and lifestyle advice based on ESC guideline recommendations (2). In addition, the control group was also offered a blood pressure monitor. The study was performed in an open design. Outcomes were assessed using objective data from two in-person visits at baseline (V0) and after an observation period of 12 weeks (V1). The CHANGE study was designed to examine how the digital health application affects therapy adherence and quality of life in 210 CCS patients. The study also aimed to provide the scientific basis for registration in the German Digital Health Applications registry, making it Germany's first refundable cardiological digital health application.

### Smartphone-guided secondary prevention digital health application (Vantis | KHK und Herzinfarkt)

The “Vantis | KHK und Herzinfarkt” digital health application is designed to support patients with CCS to accomplish recommended behavior changes based on current ESC guidelines (22). In a small pilot study, the beta version of the application was tested in patients with CCS (17). The final version covers a broader range of therapy areas and is personalized. It also includes vital monitoring through connected devices, with an upper arm blood pressure monitor being part of this study. Additionally, the application features game design and a forum where patients can exchange questions and ideas.

The digital health application provides a daily plan for the patients, which includes video-guided home exercises depending on the patient's capabilities, medication tracking and educational units in brief texts. A digital nutrition coaching program offers advice on a healthy diet according to ESC guidelines. Patients can also take their blood pressure at home and record their body weight, food intake, and any specific symptoms they experience. The digital health application tracks symptoms such as angina pectoris and vital parameters like elevated blood pressure and informs patients about these parameters. This information may include recommendations to discuss the matter during their next doctor appointment, visit a doctor immediately, or call an ambulance. The software collects data provided by patients or connected devices such as the blood pressure monitoring device Omron HEM-7155T-D used in this study. The app is designed to increase adherence and includes elements of game design to motivate patients. Positive behavior, such as completing activities within the application, is rewarded as a cornerstone of the program.

### Study population and recruitment

Patients with CCS (I25, ICD-10) aged ≥18 years were enrolled. Potential candidates were screened at the study sites for eligibility. As a second inclusion criterion, eligible patients had to possess a compatible smartphone and be capable of independent use of the device. Exclusion criteria were divided into two groups: study-related and device-related. Study-related exclusion criteria were a planned or completed participation in a CAD-related rehabilitation program within the past 2 months, participation in another clinical trial, and conflict of interest to the study's sponsor or investigator. Device-related exclusion criteria were no access to a compatible smartphone, limited capability to handle a smartphone, and insufficient language proficiency, as the digital health application is currently available only in German. Eligible patients were approached by a study physician and written informed consent was obtained. During the initial visit, before randomization, patients received some brief information on CAD, modifiable risk factors and secondary prevention measures. Recruitment started in July 2022 and was completed in April 2023.

### Study endpoints and data collection

A subgroup analysis of the original CHANGE cohort was performed to investigate the effect of the digital health application on reducing BP. Thus, three subgroups of patients with elevated baseline BP were eligible for analysis. The primary endpoint was systolic BP reduction after 12 weeks in patients with hypertensive systolic BP at baseline, defined as systolic BP ≥140 mmHg. Secondary endpoints were changes in diastolic BP in hypertensive patients with a diastolic BP ≥90 mmHg and systolic BP changes in patients with high-normal and hypertensive systolic BP at baseline, defined as systolic BP ≥130 mmHg. Patients with hypertension and BP values within the target range were also analysed. According to current ESH guidelines, the target range was defined as systolic BP <130 mmHg in patients <65 years of age and <140 mmHg in patients ≥65 years of age. Furthermore, patients with a BP journal (recorded BP derived from home measurements for at least 8 of the past 14 days) were analyzed. Office BP values were obtained during study visits V0 and V1 by trained study staff according to current guidelines of the European Society of Hypertension (23). For BP measurements, patients were asked to avoid consuming nicotine, caffeine or food and to avoid exercise 30 min before the visit. BP was measured after the patients were required to sit for 5 min; neither patients nor study staff talked during BP measurements. BP values were obtained in patients sitting upright with the back supported by the chair, with the arm resting on a table, and the mid-arm being positioned at heart level. Using an oscillometric upper arm cuff device, three BP values were obtained, and the average of the last two measurements used when the difference between those measurements in systolic BP was smaller than 5 mmHg. This method was applied once per visit. Clinical data were obtained using medical reports and charts at each study center.

### Data management

Data were managed by the clinical research organization (CRO Dr. med. Kottman GmbH & Co. KG) of the study using “SecuTrial” (iAS interActive Systems GmbH) to create an eCRF for each patient. The database system automatically generated a code for each patient to protect their identity. After this pseudonymization, the database software performed randomization in a 1:1 manner. Entered data were audited by a monitor of the CRO.

### Statistical considerations

Statistical analyses were performed independently by the CRO of the study using the current version of SAS (Statistical Analysis Software), SAS Institute Inc. Cary, NC, USA. The CHANGE study was designed as an intention-to-treat analysis. Group differences in the primary endpoint were analyzed by independent *t*-test (*p* = 0.05). Intraindividual differences between V0 and V1 were analyzed using a dependent *t*-test (*p* = 0.05). Between-group differences at V1 were analyzed using Chi-square testing (*p* = 0.05). Missing data at V1 were replaced using reference-based imputation by replacing the missing value with the control group's mean. In addition, a sensitivity analysis was performed using only data of “completers” in whom values from both V0 and V1 were available. Additional analyses for potential confounders were performed for patients with hypertension and stable medication 2 weeks before enrolment until V1 and patients with no acute cardiac event in the past 6 months. Throughout the manuscript, data are presented as means ± standard deviation.

### Ethics

This study complies with local legal requirements and has been evaluated by the ethics committees of the university of Bonn (479/21), university of Düsseldorf (2022–1868), medical chamber North-Rhine (Ärztekammer Nordrhein, 2022049) and medical chamber Rhineland-Palatinate (Ärztekammer Rheinland-Pfalz, 2022-16378) for the respective centers. The study was performed in accordance with the Declaration of Helsinki; all patients provided written informed consent to participate.

### Registration

The study is registered at the German study registry number DRKS00028081.

## Results

### Characteristics of the study patients

In total, all analyzed subgroups contained 91 patients that showed systolic BP levels BP ≥130 mmHg or diastolic BP levels ≥90 mmHg and were eligible to investigate primary and secondary endpoints regarding BP reduction (Tables 1, 2). Patients in the intervention group showed a mean usage of the digital health application in 5.9 days/week and concluded 7.4 activities per day.

**Table 1:** Baseline characteristics of patients with baseline systolic BP ≥140 mmHg.

Baseline	≥140 mmHg Sys.		
	Total	Intervention	Control
*N*	44	25	19
Age (SD)	65,1 (7,7)	66,1 (6,6)	63,7 (8,9)
Male sex (%)	82	80	84
BMI (SD)	29,2 (5,5)	29 (5,4)	29,5 (5,8)
Current smoker (%)	16	8	26
Hypertension (%)	100	100	100
Diabetes (%)	27	36	16
Dyslipidemia (%)	91	84	100
Asthma (%)	11	12	11
COPD (%)	5	4	5
Chronic heart failure (%)	25	20	32
CAD (%)	100	100	100
CAD diagnosis (months)	68,8	75,2	60,4
CAD diagnosis (%, <6 months)	11	4	21
Myocardial infarction (%)	55	48	63
Myocardial infarction (months)	57,3	57,2	57,4
Myocardial infarction (%, <6 months)	7	8	5
Coronary angiography (%)	80	80	79
Angina pectoris (%)	25	24	26
Dyspnea (%)	36	36	37
NYHA class (mean)	1,4	1,4	1,4
PTCA/Stenting (%)	75	76	74
Stable pharmacotherapy (%)	86	92	79
DMP (%)	43	32	58
Left ventricular ejection fraction (%)			
>55%	65	72	58
54–45%	30	24	34
44–35%	5	4	5
<35%	0	0	0
Systolic blood pressure mmHg (SD)	153,9 (13)	153,8 (15,8)	153,9 (8,6)
130–139 mmHg (%)	0	0	0
140–159 mmHg (%)	75	76	74
160–179 mmHg (%)	20	16	26
≥180 mmHg (%)	5	8	0
Diastolic blood pressure mmHg (SD)	88,4 (9,5)	87,4 (10,9)	89,7 (7,4)
Antihypertensive drugs (%)	100	100	100
ACE-inhibitor (%)	50	52	47
AT1-receptor-antagonist (%)	45	44	47
Calcium antagonist (%)	34	36	32
Betablockers (%)	68	64	74
Mineralocorticoid-receptor-antagonists (%)	7	4	11
Diuretic (%)	32	36	26
Others (%)	11	16	5
Therapy regime (%)			
No (%)	0	0	0
Mono (%)	16	12	21
Dual (%)	39	40	37
Triple (%)	37	32	21
More than 3 (%)	18	16	21
Number of antihypertensive drugs (mean)	2.5	2.5	2.4

DMP, disease-modifying program.

**Table 2:** Baseline characteristics of patients with baseline systolic BP ≥130 mmHg or diastolic BP ≥90 mmHg.

Baseline	≥130 mmHg Sys.			≥90 mmHg Dia.		
	Total	Intervention	Control	Total	Intervention	Control
*N*	89	44	45	28	9	19
Age (SD)	64,2 (8,5)	65,7 (7,8)	62,7 (9,1)	62,4 (8,4)	66,1 (4,1)	60,6 (9,4)
Male sex (%)	83	80	87	96	89	100
BMI (SD)	29 (5,2)	28,7 (5,6)	29,3 (5)	30,3 (4,6)	30,5 (5,9)	30,2 (4)
Current smoker (%)	17	11	22	21	22	21
Hypertension (%)	99	100	98	90	90	90
Diabetes (%)	29	32	27	18	22	16
Dyslipidemia (%)	91	89	94	86	78	89
Asthma (%)	10	10	11	7	0	11
COPD (%)	7	5	9	7	10	5
Chronic heart failure (%)	37	32	42	40	33	42
CAD (%)	100	100	100	100	100	100
CAD diagnosis (months)	71,2	64,8	77,5	63,5	48,3	70,7
CAD diagnosis (%, <6 months)	10	9	11	7	11	5
Myocardial infarction (%)	57	48	67	64	33	74
Myocardial infarction (months)	68,6	61,5	73,7	44,2	25,0	49,7
Myocardial infarction (%, <6 months)	3	5	2	7	22	0
Coronary angiography (%)	81	84	80	86	89	79
Angina pectoris (%)	26	32	20	18	22	16
Dyspnea (%)	35	34	36	32	33	32
NYHA class (mean)	1,5	1,5	1,6	1,5	1,6	1,4
PTCA/Stenting (%)	81	80	82	82	78	84
Stable pharmacotherapy (%)	84	82	87	89	89	90
DMP (%)	26	30	22	21	33	16
Left ventricular ejection fraction (%)						
>55%	58	61	57	71	7	68
54–45%	12	27	31	21	2	21
44–35%	13	7	9	4	0	5
<35%	2	0	4	0	0	0
Mean systolic blood pressure mmHg (SD)	143,9 (13,6)	145,6 (15,2)	142,2 (11,7)	147,1 (18,4)	160,2 (23,8)	140,9 (11,4)
130–139 mmHg (%)	51	45	58	32	11	42
140–159 mmHg (%)	36	41	31	46	44	47
160–179 mmHg (%)	10	9	11	11	22	0
≥180 mmHg (%)	2	5	0	11	22	0
Mean diastolic blood pressure mmHg (SD)	85,5 (9,6)	84,4 (10,3)	86,7 (8,8)	95,6 (7,2)	97,9 (8,3)	94,5 (6,5)
Antihypertensive drugs (%)	98	98	98	96	89	100
ACE-inhibitor (%)	45	48	42	50	44	53
AT1-receptor-antagonist (%)	40	43	38	32	44	26
Calcium antagonist (%)	27	27	27	29	33	26
Betablockers (%)	61	57	64	68	44	79
Mineralocorticoid-receptor-antagonists (%)	9	7	11	18	11	21
Diuretic (%)	30	30	31	32	44	26
Others (%)	7	9	4	14	33	5
Therapy regime (%)						
No (%)	2	2	2	4	11	0
Mono (%)	24	18	29	21	11	26
Dual (%)	39	45	33	25	22	26
Triple (%)	22	25	20	29	22	32
More than 3 (%)	10	7	13	18	22	16
Number of antihypertensive drugs (mean)	2.2	2.2	2.2	2.4	2.6	2.4

DMP, disease modifying program.

For the primary endpoint, 44 patients had a systolic BP ≥140 mmHg with a mean of 153.9 mmHg at baseline (Table 1). The mean age in this group was 65 years, and the majority of the patients were male (82%). In the control group, more patients were identified to be smokers than in the intervention group (26% vs. 8%). All patients with BP levels ≥140 mmHg at baseline had a preexisting diagnosis of hypertension and, thus, insufficient BP control despite established pharmacological therapy in all patients. The mean number of drugs for antihypertensive treatment in this group was 2.5. 24% of patients in the intervention group and 21% of patients in the control group received SGLT2-inhibitors at baseline whereas 11% of patients in the control group and 0 patients in the intervention group received GLP-1 receptor agonists at baseline. Medication with these two substances remained unchanged between V0 and V1. Clinical symptoms of CAD were balanced between groups, with angina pectoris in 25% and exertional dyspnea in 36% of patients. However, more patients in the control group had a CAD diagnosis for a shorter period than 6 months (21% vs. 4%). More than half of the patients had experienced myocardial infarction.

45 patients had a systolic BP of 130–139 mmHg at baseline, classified as “high-normal” BP in addition to 44 patients with hypertensive BP. The mean systolic BP in the combined group of patients with hypertensive and high-normal BP was 143.9 mmHg (Table 2). Among those patients, 99% had a preexisting diagnosis of hypertension. Patients in the intervention group were older (65.7 vs. 62.7 years) and less likely to have experienced a myocardial infarction in the past (48% vs. 67%).

28 patients had a diastolic BP ≥90 mmHg at baseline. In total, these patients appeared to be younger with a higher BMI compared to the other groups. However, patients in the intervention group were older (66.1 vs. 60.6 years) than the control group. 90% of patients in both groups had a pre-existing diagnosis of hypertension at baseline.

### Primary endpoint

**Figure 1:**
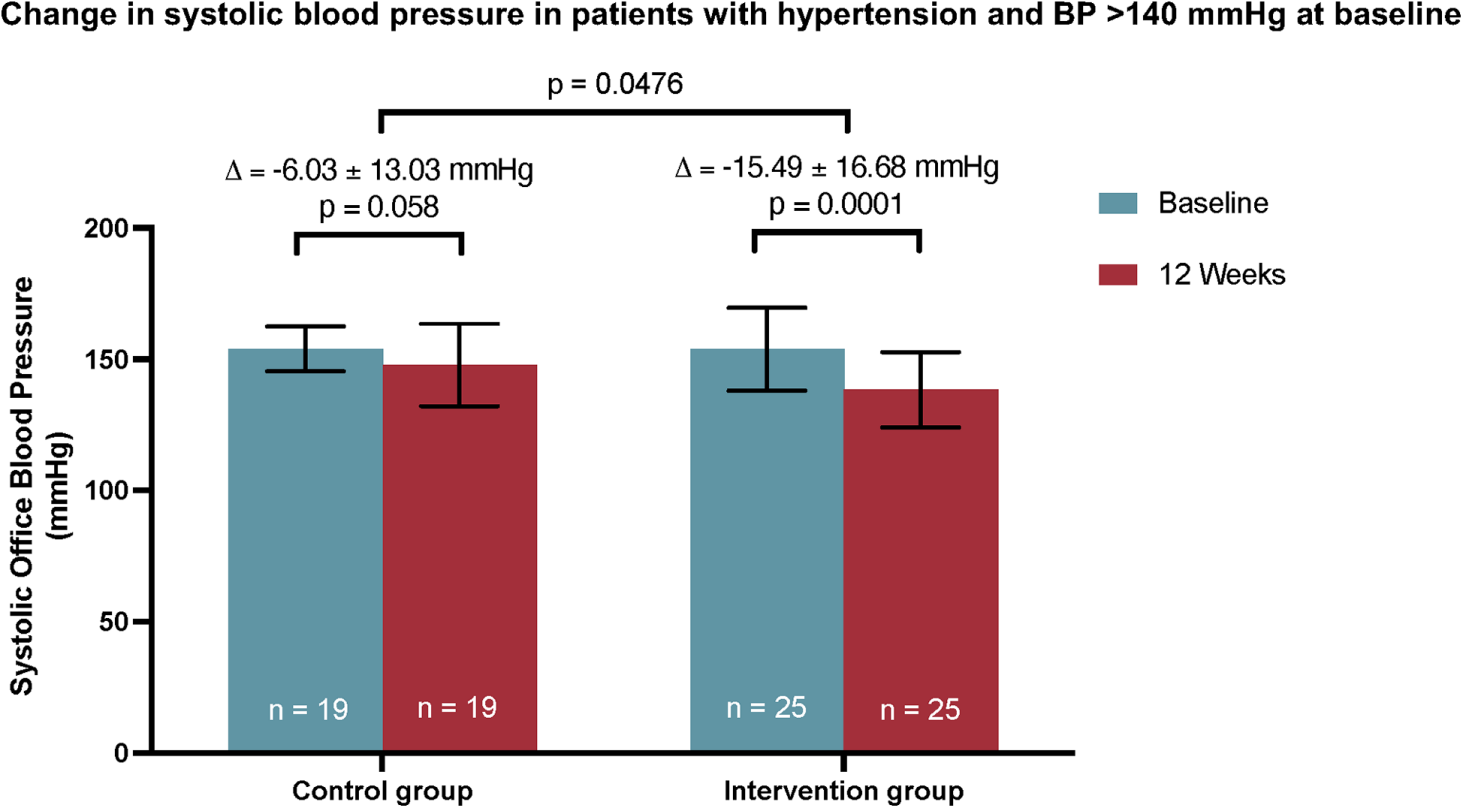
The primary endpoint of the subgroup analysis of the CHANGE study. A significant reduction in systolic BP was observed only in the intervention group using Vantis | KHK und Herzinfarkt in addition to standard care. The between-group difference achieved statistical significance. Mean ± SD. Between-group differences in the primary endpoint were analyzed by independent *t*-test. Intraindividual differences between V0 and V1 were analyzed using a dependent *t*-test.

In patients with a systolic BP ≥140 mmHg at baseline, a reduced office systolic BP was observed at the end of the observation period in both the control and the intervention group (Figure 1). While the intervention group demonstrated a statistically significant reduction in BP (−15.49 ± 16.68 mmHg, *p* = 0.0001), no statistically significant difference in systolic BP was observed in the control group (−6.03 ± 13.03 mmHg, *p* = 0.058). Furthermore, the between-group difference in systolic BP reduction was statistically significant (*p* = 0.048). Thus, the digital health application, in combination with standard care, showed a significant effect on BP reduction compared to standard care alone in this subgroup of patients.

### Secondary endpoints

**Figure 2:**
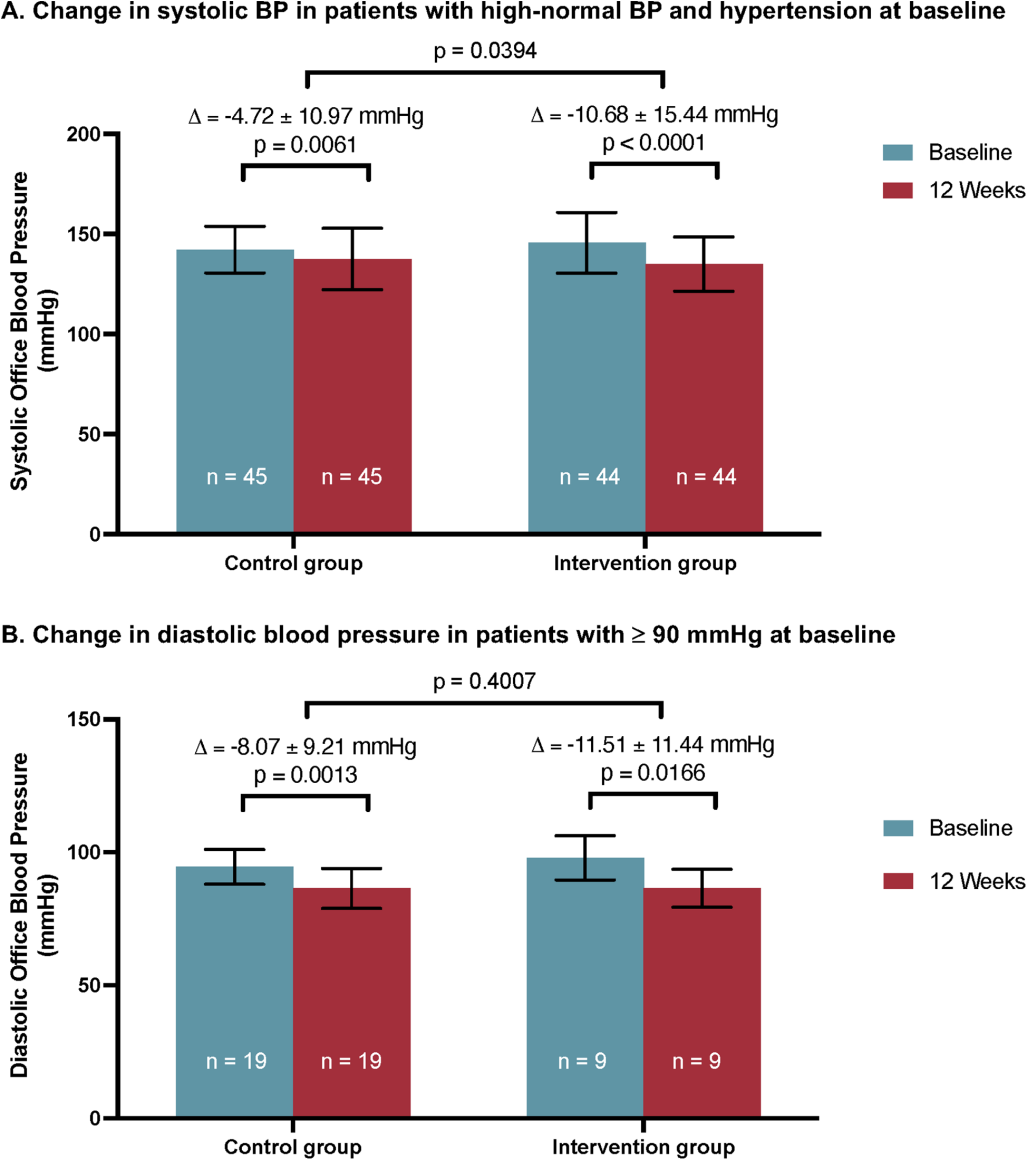
Secondary endpoints of the CHANGE Study. **(A)** Significant reduction in systolic BP in patients with high-normal and hypertensive BP at baseline was observed in the intervention and control groups. The between-group difference was statistically significant. Mean ± SD. **(B)** Reduction in diastolic BP in patients with diastolic BP of ≥90 mmHg at baseline was significant in both groups. The between-group difference was not statistically significant. Mean ± SD. Between-group differences were analyzed by independent *t*-test. Intraindividual differences between V0 and V1 were analyzed using a dependent *t*-test.

Patients with a systolic BP of ≥130 mmHg at baseline exhibited lower systolic BP values at V1 compared to V0 in both groups (Figure 2A). In this combined group, BP was significantly decreased at V1 in the intervention group (−10.68 ± 15.44 mmHg, *p* < 0.0001). A less pronounced, albeit statistically significant reduction of systolic BP was also observed in the control group (−4.72 ± 10.97 mmHg, *p* = 0.0061). In the between-group comparison, the systolic BP reduction at V1 in the intervention group was again significantly greater than in the control group (*p* = 0.039). Therefore, the intervention group showed a significant improvement in BP reduction compared to the control group in patients with high-normal and hypertensive BP values at baseline.

Further, the effect of the intervention on diastolic BP was investigated in patients with a diastolic BP ≥90 mmHg at baseline (Figure 2B). Both groups showed a significant reduction in diastolic BP after 12 weeks (−11.51 ± 11.44 mmHg, *p* = 0.0166 in the intervention group vs. −8.07 ± 9.21 mmHg, *p* = 0.0013 in the control group). The difference between groups was not statistically significant (*p* = 0.4).

Patients with a systolic BP ≥140 mmHg at baseline were further analyzed to determine whether they achieved the target BP value after the observation period of 12 weeks (Table 3). The intervention group achieved a numerically higher rate of patients within the target range at V1 than the control group (48% vs. 26%). However, this observation did not reach statistical significance between groups (*p* = 0.1434). Patients with an indication for home BP monitoring using a BP journal (*n* = 43) were analyzed regarding their adherence to a BP journal. Significantly more patients in the intervention group had documented BP values for more than 8 of the past 14 days compared to the control group (64.0% vs. 27.8%, *p* = 0.0191).

**Table 3:** Patients with systolic BP values within the target range at V1 and analysis of patients with documented home BP values (≥8 days of the past 14 days).

	Control group		Intervention group		*P*
	V1 (%)	*N*	V1 (%)	*N*	
Blood pressure within target range	5 (26)	19	12 (48)	25	0.1434
Blood pressure journal ≥8 days in the past 14 days	5 (27)	18	16 (64)	25	0.0191

Chi-square testing.

### Further analyses

We analysed patients completing the study with data available from V0 and V1 (completers). This group consisted of 35 patients with systolic BP ≥140 mmHg at baseline, with 20 patients in the intervention and 15 in the control group, respectively (Table 4). The primary endpoint findings were consistent in this subgroup, with a significantly greater BP reduction in the intervention group (*p* = 0.046).

**Table 4:** Subgroup analyses of patients with systolic BP ≥140 mmHg at baseline.

Subgroup	Change in systolic blood pressure				
	*N*	Mean	SD	CI	*p*
Systolic blood pressure ≥140 mmHg					
IG	25	−15.5	16.7	−22.4, −8,6	<0.001
Between-group difference (IG vs. CG)	44	−9.5	15.2	−18.8, −0.1	0.048
Completer					
IG	20	−17.9	17.9	−26.2, −9.5	<0.001
Between-group difference (IG vs. CG)	35	−11.8	16.7	−23.4, −0.2	0.046
Stable pharmacotherapy					
IG	23	−15.8	17.3	−23.2, −8.3	<0.001
Between-group difference (IG vs. CG)	38	−9.8	15.9	−20.5, 0.2	0.071

Between-group differences in the primary endpoint were analyzed by independent *t*-test. Intraindividual differences between V0 and V1 were analyzed using a dependent *t*-test. CG, control group; IG, intervention group; SD, standard deviation.

Pharmacological therapy is a main pillar in the treatment of hypertension. Details about antihypertensive medication are displayed in Tables 1, 2. All patients with a systolic BP ≥140 mmHg received antihypertensive medication and changes in antihypertensive medication were permitted by the study protocol. The majority were taking dual drug therapy in both the intervention and the control group (40% vs. 37%). As changes in antihypertensive medication during the study might be potent confounders, we analyzed a subset of patients with systolic BP ≥140 mmHg and a stable medication 2 weeks before enrolment until V1. This subset included 38 patients (86%). In this group, the reduction of systolic BP in the intervention group was consistent (15.8 ± 17.3 mmHg, *p* < 0.001). However, in this small subgroup, the between-group difference was not signficant (*p* = 0.071). In patients with systolic BP ≥130 mmHg and stable pharmacotherapy, reduction of systolic BP was consistent (intervention group: 12.1 ± 15.6 mmHg, *p* < 0.001) with a statistically significant between-group difference (*p* = 0.025) (Table 5). The subgroup of patients with diastolic BP ≥90 mmHg with stable pharmacotherapy showed consistent results without a significant between-group difference.

**Table 5:** Subgroup analyses of systolic BP reduction in patients with systolic BP ≥130 mmHg at baseline and diastolic BP reduction in patients with diastolic BP ≥90 mmHg.

Subgroup with stable pharmacotherapy	Change in systolic blood pressure				
	*N*	Mean	SD	CI	*p*
Systolic blood pressure ≥130 mmHg					
IG	36	−12.1	15.6	−17.4, −6.8	<0.001
Between-group difference (IG vs. CG)	75	−7.3	13.5	−13.4, −1.1	0.0245
Subgroup with stable pharmacotherapy	Change in diastolic blood pressure				
	*N*	Mean	SD	CI	*p*
Diastolic blood pressure ≥90 mmHg					
IG	8	−12.7	11.62	−22.4, −3.0	0.0176
Between-group difference (IG vs. CG)	25	−3.8	10.7	−12.9, 5.4	0.4347

Between-group differences were analyzed by independent *t*-test. Intraindividual differences between V0 and V1 were analyzed using a dependent *t*-test. CG, control group; IG, intervention group; SD, standard deviation.

## Discussion

In the present study, we report that incorporating a multicomponent digital health application guided by a smartphone in addition to standard care can effectively improve BP levels in CCS patients with hypertension and insufficient BP control. We observed a significantly greater reduction in systolic BP in the intervention group as compared to patients treated with standard care alone. This effect was consistent in a combined subgroup of patients with high-normal BP and hypertension.

Modifiable cardiovascular risk factors are major contributors to global morbidity and mortality. Large-metanalyses suggest that approximately 22% and 19% of deaths from any cause among women and men are attributable to five modifiable cardiovascular risk factors, including systolic BP (24). The risk of morbidity and premature death indicated by these factors is further aggravated in patients with cardiovascular disease (25). Therefore, optimising modifiable cardiovascular risk factors is a key secondary prevention component, especially for patients with CCS (2). Major aspects of risk factor management are lifestyle changes and pharmacological therapy to slow the progression of CCS and improve overall prognosis (2). However, one of the main obstacles to achieving optimal results is poor adherence to medication and lifestyle recommendations (11). Recent data suggest that after experiencing a cardiovascular event, 70% of patients fail to make the necessary adjustments in their behaviour to reduce the risk of future incidents effectively (4, 11). Additionally, only 40% of individuals with CAD reduced their intake of saturated fats despite efforts to improve their dietary habits. In this context, E-Health approaches that use apps have positively influenced behavioural realignment (17, 19, 21, 26).

In addition to drug therapy, lifestyle interventions have been proven effective and are thus a mainstay of risk factor management (27). Studies have shown that physical exercise can decrease systolic BP by up to 20 mmHg in people with resistant hypertension (28, 29). Another study performed in patients with resistant hypertension showed that 12 weeks of moderate-intensity exercise training decreased 24-hour systolic BP by 7.1 mmHg and office BP by 10 mmHg compared to standard care (30). Another important aspect of lifestyle interventions is a healthy diet. ESC guidelines on CCS advocate adhering to a Mediterranean diet, which consists of consuming a high proportion of fruits, vegetables, fish, and nuts while reducing the intake of red meat and saturated fats (2, 16). In a study performed in a cohort of 55–80-year-old patients in Spain, higher consumption of nuts or olive oil significantly decreased diastolic blood pressure compared to a low-fat diet (31). In another study, adherence to a Mediterranean diet for 1 year decreased systolic BP by 5.5 mmHg without significant changes in diastolic BP compared to control (32). In our study, the intervention group demonstrated a significant and clinically relevant decrease in systolic BP of 15.5 mmHg, which was significantly enhanced compared to standard care alone. The digital health application investigated in this study is a multicomponent intervention based on ESC guidelines. The combination of digital nutrition advice, a medication memory function, and video-guided home exercises aims to increase adherence and endorse lifestyle modifications.

Our study has limitations, such as its open design. Another limitation is the relatively small sample size of patients eligible for analysis of the primary endpoint, as BP measurements can vary depending on the measurement methods, even when strictly adhering to guideline recommendations. Only a subgroup of the randomized original cohort was analyzed, which affects the statistical power of our analyses. Furthermore, this results in differently sized cohorts eligible for investigating the primary endpoint (19 vs. 25 patients in the intervention and control group). However, despite the small sample size, we detected a significant improvement in systolic BP with the described intervention within the relatively short observation period of 12 weeks. In the small subgroup of patients with systolic BP ≥140 mmHg and stable pharmacotherapy at V0 and V1 the between-group difference of systolic BP reduction was not statistically significant. However, this observation may be due to the small sample size of this subgroup as the intervention group showed statistically significant greater reduction in systolic BP in patients with systolic BP ≥130 mmHg at baseline and stable pharmacotherapy. As the investigated digital health application is a multifactorial intervention, the study design allows no conclusion on which the effect of BP reduction is based on. Between-group differences in preexisting comorbidities might be a confounder as patients with diabetes mellitus or heart failure and CCS are likely to be more closely monitored by physicians. Last, as the investigated intervention requires access to a smartphone and adequate language proficiency, a selection bias might occur to a less deprived population of patients with CCS in this study.

The strengths of the study are its prospective and randomized character. The study design prevents the influence of potential confounders such as participation in a cardiac rehabilitation program. The multi-component digital health application is designed based on ESC guidelines and promotes a multidisciplinary approach to managing lifestyle. It addresses various risk factors and encourages patients to actively participate in secondary prevention measures (2).

## Conclusion

According to the CHANGE study, we hypothesize that the use of the investigated smartphone digital health application, in addition to standard care, has a positive effect on reducing BP in patients with CCS who have inadequate BP control. This observation could improve secondary prevention measures for a high-risk population. Our study underlines the great potential of the rapidly evolving field of digital cardiology. Nevertheless, the effects on cardiovascular risk factors and prognostic endpoints, such as cardiovascular events and mortality, still need to be investigated in larger, appropriately powered studies.

## Data Availability

The raw data supporting the conclusions of this article will be made available by the authors, upon reasonable request.
